# Prevalence and cognitive factors influencing high-risk HPV infection and cervical diseases in women aged 18–45 in Shijiazhuang city

**DOI:** 10.1097/MD.0000000000041436

**Published:** 2025-02-21

**Authors:** Xiaohui Deng, Yonghong Yang, Xiaoqing Pang, Xiaoyan Wen, Zhengyan Dai

**Affiliations:** a Gynecology Department, Shijiazhuang Maternal and Child Health Hospital, Shijiazhuang, Hebei, China; b Prenatal Diagnosis Department, Shijiazhuang Maternal and Child Health Hospital, Shijiazhuang, Hebei, China; c Ultrasound Treatment Outpatient Department, Shijiazhuang Maternal and Child Health Hospital, Shijiazhuang, Hebei, China.

**Keywords:** Shijiazhuang city, high-risk human papillomavirus, cervical diseases, current situation, awareness

## Abstract

This study aimed to investigate the prevalence of high-risk human papillomavirus (HPV) infection and the awareness levels regarding cervical diseases among women aged 18–45 in Shijiazhuang city. The objectives were to determine the incidence rates of high-risk HPV infections, analyze the patterns of cervical disease occurrence, and identify the factors influencing awareness within this demographic. A total of 544 women aged 18–45 participated in the study, with 102 testing positive for high-risk HPV infection. A structured questionnaire was administered to evaluate awareness of high-risk HPV and cervical diseases. The survey collected data on infection prevalence, subtype distribution, incidence rates, knowledge levels, and factors affecting awareness related to high-risk HPV infections and cervical health. Among the 544 women screened, 102 (18.75%) were diagnosed with high-risk HPV. HPV-16 emerged as the most prevalent subtype, followed by HPV-52 and HPV-58. Of the positive cases, 38 displayed no signs of intraepithelial neoplasia or malignant lesions, while 38 had atypical squamous epithelium, predominantly associated with HPV-52. Low-grade intraepithelial neoplasia was observed in 15 cases, and high-grade neoplasia was found in 11 cases, both primarily linked to HPV-16. Awareness levels varied, with 87 participants demonstrating low knowledge and 15 showing higher awareness. Logistic regression analysis identified education, occupation, residence, and access to scientific knowledge as significant factors influencing awareness and infection risk (*P* < .05). The prevalence of high-risk HPV infection among women aged 18–45 in Shijiazhuang city is relatively low, with HPV-16 being the predominant subtype. HPV-16 was strongly associated with cervical epithelial neoplasia and cervical cancer. Targeted educational interventions, particularly for populations with lower education levels and those in rural areas, are recommended to enhance awareness and improve the prevention and control of HPV-related infections and cervical diseases.

## 1. Introduction

Human papillomavirus (HPV) is a small, double-stranded DNA virus with over 100 subtypes, commonly associated with reproductive tract infections.^[1,2]^ Most HPV infections are transient and asymptomatic, with the immune system clearing the virus within 2 to 24 months, resulting in minimal impact on most women’s quality of life.^[3,4]^ However, approximately 15% of HPV infections persist, particularly those caused by high-risk subtypes. These persistent infections integrate viral DNA into the host genome, allowing the virus to evade immune responses and disrupt cellular regulation.^[5,6]^ High-risk HPV infections are strongly linked to squamous metaplasia, tumor suppressor gene inactivation, and oncogene overexpression, making them a major risk factor for cervical cancer and precancerous lesions. Given these risks, enhancing the early prevention and control of high-risk HPV infections is crucial for reducing cervical cancer incidence and progression, aligning with the goals outlined in the China Women’s Development Outline (2011–2020).^[7–9]^

Recent advancements in medical technology have enabled the combination of HPV-DNA and cervical cytology testing, addressing the limitations of traditional exfoliative cytology methods, such as low sensitivity and high false-negative rates. This combined testing approach has proven valuable for early detection and prevention of HPV-related diseases, including cervical cancer.^[7,8]^ However, many women remain reluctant to undergo HPV-DNA testing, contributing to suboptimal cervical cancer control rates in China. This reluctance is largely attributed to insufficient awareness of HPV infections and cervical diseases among women.^[10–13]^

With the shift towards preventive medicine, the focus of disease intervention has transitioned from treatment to prevention and control. Understanding the current prevalence of high-risk HPV infections and cervical diseases, along with the associated cognitive risk factors, is critical for designing targeted interventions. Nevertheless, research over the past 3 years on the prevalence and awareness of high-risk HPV infections and cervical diseases in local populations has been limited. This lack of data restricts a comprehensive understanding of the issue and hinders the development of evidence-based prevention strategies.

This study aims to address this gap by investigating the prevalence of high-risk HPV infections and cervical diseases among women aged 18–45 in Shijiazhuang city. Specifically, we hypothesize that awareness levels regarding high-risk HPV infections and cervical diseases are significantly associated with factors such as education level, occupation, and access to scientific knowledge. Furthermore, we posit that improving awareness can effectively reduce infection risks and enhance early prevention efforts.

The findings of this study will provide a foundation for developing evidence-based prevention strategies and serve as a guide for cervical cancer screening and management in this region.

## 2. Materials and methods

### 2.1. Data source

This study was approved by the Ethics Committee of Shijiazhuang Maternal and Child Health Hospital (Ethics Approval No. 2020n350). The sample comprised 544 women aged 18–45 who underwent physical examinations in the local area between January 2020 and March 2021. Participants were selected using a stratified random sampling method to ensure representativeness across urban and rural areas. Among the participants, 51.1% (278 women) resided in urban areas, and 48.9% (266 women) were from rural areas, reflecting the demographic distribution of the region. This sampling strategy was designed to balance the proportion of rural and urban participants, thereby enhancing the generalizability of the study findings.

Among the 544 participants, 417 were married, and 127 were unmarried, with ages ranging from 23 to 44, as shown in Table 1. Education levels varied: 53 participants had an education level of junior high school or below, 176 had a high school education, and 315 had a college degree or above. Regarding sexual history, 495 participants reported having 1 sexual partner, while 49 reported having 2 or more. Additionally, 23 participants reported a family history of cervical cancer, 405 had a history of childbirth, and 139 had experienced a miscarriage.

**Table 1:** Demographic and social characteristics of the sample of women (N = 544).

Characteristic	Category	N	Percentage (%)
Age	<25 yr	43	7.91%
	25–30 yr	31	5.70%
	>30 yr	470	86.39%
Marriage status	Married	417	76.6%
	Unmarried	127	23.4%
Education level	Junior high school or below	53	9.7%
	High school	176	32.3%
	College degree or above	315	58.0%
Occupation	Administrative institutions/enterprises	77	14.1%
	Company employees	20	3.7%
	Self-employed/individual	21	3.9%
	Gig worker/other	426	78.3%
Residence	Urban	278	51.1%
	Rural	266	48.9%
Household income (monthly)	>5000 yuan/mo	53	9.7%
	≤5000 yuan/mo	491	90.3%
Family history of cervical cancer	Yes	23	4.2%
	No	521	95.8%
History of childbirth	Yes	405	74.5%
	No	139	25.5%
History of miscarriage	Yes	139	25.5%
	No	405	74.5%

The study strictly adhered to ethical guidelines, emphasizing the protection of personal privacy and obtaining informed consent from all participants. Each participant was informed about the study’s purpose, methods, and significance and was assured of their right to withdraw from the study at any time without consequences.

The inclusion criteria were as follows: residency or study in the local area for more than 6 months, female participants aged 18–45, good overall health with normal organ function, a history of sexual activity, non-menstrual period during the study, and the ability to comprehend, communicate, and engage in self-care. Exclusion criteria included having malignant tumors of other origins, a history of cervical disease or surgery, recent vaginal flushing or medication use, and recent pelvic radiation therapy or hormone use.

### 2.2. Methods

The study conducted a comprehensive literature review using Chinese databases, including Wanfang and VIP, to identify relevant factors influencing awareness and align with epidemic investigation standards. Building on clinical trial research methodologies, an improved questionnaire was developed to address sensitive topics related to women’s health and family circumstances. Expert consultations were sought to refine the questionnaire, culminating in the final version titled “Questionnaire on the Status and Risk Factors of High-Risk Human Papillomavirus (HPV) Infection and Cervical Disease in Women.”

The questionnaire encompassed key areas, including the prevalence and subtype distribution of high-risk HPV among women of childbearing age, liquid-based cytological testing, and cervical epithelial neoplasia. It also explored awareness levels, incidence rates, and risk factors associated with cervical cancer and related conditions.

Data collection was conducted by trained personnel through face-to-face, one-on-one interviews to ensure accuracy and consistency in the responses. This approach was designed to maximize participant comfort and data reliability while addressing the sensitive nature of the subject matter.

### 2.3. Statistical analysis

SPSS 21.0 software was used to perform data analysis in this study. The observational data primarily consisted of count data, which were presented as the number of cases and corresponding percentages (%). Univariate analysis was conducted using the *χ*² test, while multivariate analysis was performed using logistic stepwise regression. A significance level of α = 0.05 was applied, with *P*-values <.05 considered statistically significant.

## 3. Result

### 3.1. Infection rates and subtype distribution of high-risk HPV among women aged 18–45

Among the 544 women aged 18–45 included in the study, 102 were diagnosed with high-risk HPV infection, corresponding to an infection rate of 18.75%. The most prevalent subtype was HPV-16, followed by HPV-52 and HPV-58. Other subtypes identified included HPV-56, HPV-39, HPV-33, HPV-51, HPV-18, HPV-31, HPV-68, HPV-59, HPV-35, and HPV-45, as detailed in Table 2.

**Table 2:** Distribution of high-risk HPV subtypes.

Distribution	Example count	Composition ratio (%)	Distribution	Example count	Composition ratio (%)
HPV-16	23	22.55	HPV-51	7	6.86
HPV-18	6	5.88	HPV-52	15	14.71
HPV-31	6	5.88	HPV-56	8	7.84
HPV-33	7	6.86	HPV-58	12	11.76
HPV-35	2	1.96	HPV-59	3	2.94
HPV-39	8	7.84	HPV-68	4	3.92
HPV-45	1	0.98			

### 3.2. Distribution of high-risk HPV subtypes in cervical-related diseases

The distribution of high-risk HPV subtypes in cervical-related diseases is summarized in Table 3. Liquid-based thin-layer cytological detection was performed on 102 individuals infected with high-risk HPV. Among these cases, 38 showed no intraepithelial neoplasia or malignant lesions, 38 exhibited atypical squamous epithelium of undetermined significance, 15 had low-grade intraepithelial neoplasia, and 11 presented with high-grade intraepithelial neoplasia.

**Table 3:** Distribution of high-risk HPV infection subtypes in cervical-related diseases [cases (%)].

HPV infection subtypes	Example count	Nointraepithelial neoplastic or malignant lesions (38 cases)	Unclear atypical squamous epithelium (38 cases)	Low-grade intraepithelial neoplastic lesions (15 cases)	Highly intraepithelial neoplastic lesions (11 cases)
HPV-16	23	4 (10.53)	7 (18.42)	6 (40.00)	6 (54.55)
HPV-18	6	2 (5.26)	3 (7.89)	1 (6.67)	0 (0.00)
HPV-31	6	3 (7.89)	1 (2.63)	0 (0.00)	2 (18.88)
HPV-33	7	3 (7.89)	2 (5.26)	0 (0.00)	2 (18.88)
HPV-35	2	1 (2.63)	1 (2.63)	0 (0.00)	0 (0.00)
HPV-39	8	3 (7.89)	5 (13.16)	0 (0.00)	0 (0.00)
HPV-45	1	0 (0.00)	1 (2.63)	0 (0.00)	0 (0.00)
HPV-51	7	3 (7.89)	3 (7.89)	1 (6.67)	0 (0.00)
HPV-52	15	8 (21.05)	5 (13.16)	2 (13.33)	0 (0.00)
HPV-56	8	4 (10.53)	2 (5.26)	2 (13.33)	0 (0.00)
HPV-58	12	4 (10.53)	5 (13.16)	3 (20.00)	0 (0.00)
HPV-59	3	2 (5.26)	1 (2.63)	0 (0.00)	0 (0.00)
HPV-68	4	1 (2.63)	2 (5.26)	0 (0.00)	1 (9.09)

For cases with no neoplastic or malignant lesions, HPV-52 was the most prevalent subtype, followed by HPV-16, HPV-56, HPV-58, HPV-31, HPV-33, and HPV-51. Among individuals with atypical squamous epithelium of undetermined significance, HPV-16 had the highest proportion, followed by HPV-52, HPV-58, HPV-39, HPV-18, and HPV-51. In cases of low-grade intraepithelial neoplasia, HPV-16 remained the most prevalent subtype, followed by HPV-58, HPV-52, and HPV-56. Finally, high-grade intraepithelial neoplasia was most frequently associated with HPV-16, followed by HPV-31 and HPV-33.

### 3.3. Univariate analysis of factors affecting awareness of high-risk HPV infection and cervical diseases among women aged 18–45

The univariate analysis of factors influencing awareness of high-risk HPV infection and cervical diseases among 102 women aged 18–45 is summarized in Table 4. The awareness assessment included questions covering knowledge of HPV and cervical diseases, infection risk factors, early detection methods, vaccine administration, and prevention and control strategies. Each question was scored as 0 for incorrect responses and 1 for correct responses, with a maximum possible score of 25. The total awareness scores ranged from 0 to 25, with an average score of 15 and a median score of 16. Due to the skewed distribution of scores, participants were categorized into high and low awareness groups based on the median.

**Table 4:** Single factor analysis of the impact of high-risk HPV infection and cervical disease awareness [cases (%)].

Influence factor	High awareness (15 cases)	Low awareness (87 cases)	*X*^2^ value	*P*
Age				
<25 yr old	1 (6.67)	42 (48.28)	15.187	<.01
25–30 yr old	4 (26.67)	27 (31.03)		
>30 yr old	10 (66.67)	18 (20.69)		
Marriage				
Married	12 (80.00)	22 (25.29)	17.234	<.01
Unmarried	3 (20.00)	65 (74.71)		
Educational level				
Junior high school and below	0 (0.00)	49 (56.32)	32.336	<.01
High school	4 (26.67)	28 (32.18)		
College degree or above	11 (73.33)	10 (11.49)		
Occupation				
Administrative undertakings	9 (60.00)	8 (9.20)	30.745	<.01
Company	5 (33.33)	15 (17.24)		
Individual	1 (6.67)	20 (22.99)		
Gig worker	0 (0.00)	26 (29.89)		
Other	0 (0.00)	18 (20.69)		
Residence				
Town	13 (86.67)	38 (43.68)	9.457	<.05
Country	2 (13.33)	49 (56.32)		
Household income				
>5000 yuan/mo	14 (93.33)	39 (44.83)		<.01
≤5000yuan/mo	1 (6.67)	48 (55.17)		
Popularization of science knowledge				
High penetration rate	15 (100.00)	16 (18.39)	36.513	<.01
Insufficient popularization	0 (0.00)	71 (81.61)		

Univariate analysis revealed statistically significant differences between the high and low awareness groups for factors including age, marital status, education level, occupation, place of residence, family income, and access to scientific knowledge (*P* < .05).

### 3.4. Multivariate logistic stepwise regression analysis of factors influencing awareness of high-risk HPV infection and cervical diseases among women aged 18–45

A multivariate logistic stepwise regression model was developed to examine factors influencing awareness of high-risk HPV infection and cervical diseases among women of childbearing age. Awareness level served as the dependent variable, while age, marital status, education level, occupation, place of residence, family income, and access to scientific knowledge were included as independent variables. The backward selection method was used, with exclusion criteria set at α = 0.10 and inclusion criteria at α = 0.05.

The dummy variable design for the dependent and independent variables is presented in Table 5. Logistic regression analysis identified low education level, specific occupations (individual, gig work, and other categories), rural residence, and insufficient access to scientific knowledge as significant adverse factors affecting awareness of high-risk HPV infection and cervical diseases (*P* < .05). Detailed results are provided in Table 6.

**Table 5:** Assignments of relevant factors influencing high-risk HPV infection and cervical disease awareness.

Variable name	Variable	Assignment
Awareness	Y	0 = high awareness, 1 = low awareness
Age	X1	0 = >30 yr old, 1 = ≤30 yr old
Marriage	X2	0 = married, 1 = unmarried
Educational level	X3	0 = college or above, 1 = high school or below
Occupation	X4	0 = administrative institutions, enterprises, 1 = individuals, gigs, and others
Residence	X5	0 = urban, 1 = rural
Household income	X6	0 = >5000 yuan/mo, 1 = ≤5000 yuan/mo
Popularization of science knowledge	X8	0 = high penetration rate, 1 = insufficient penetration rate

**Table 6:** Multivariate logistic stepwise regression model analysis of the impact of high-risk cervical HPV infection and cervical disease awareness in women aged 18–45.

Indicators/factors	Regression coefficient	Standard error	Wald *X*^2^ value	*P*	OR value	95% CI
Constant	0.134	0.072	3.422	.064	–	–
Constant	0.880	0.404	4.747	<.05	2.411	1.029–5.321
Educational level	0.800	0.279	8.211	<.05	2.226	1.288–3.848
Occupation	0.299	0.139	4.638	<.05	1.349	1.027–1.771
Residence	1.159	0.277	17.450	<.01	3.186	1.850–5.487

## 4. Discussion

The findings of this study indicate that 18.75% of women of childbearing age in the region have high-risk HPV cervical infections, a prevalence lower than that reported in existing literature.^[14]^ Among women aged 18–45 in this area, the most common subtypes identified were HPV-16, HPV-52, and HPV-58, consistent with previous epidemiological studies.^[15–17]^ This subtype distribution suggests stability in the local epidemiological pattern, providing valuable insights for vaccine development, vaccination strategies, and clinical prevention efforts. However, the elevated incidence of HPV-52 and HPV-58 warrants further clinical attention and research.

The study also revealed that different high-risk HPV subtypes are linked to specific cervical epithelial lesions. For instance, HPV-16 was predominantly associated with atypical squamous epithelial and intraepithelial neoplasia, highlighting its critical role in cervical pathology. These findings emphasize the importance of subtype-specific prevention and control measures to address high-risk HPV infections effectively.

This study further assessed awareness and influencing factors related to high-risk HPV infection and cervical diseases among women of childbearing age. The results demonstrated a notable lack of awareness within the local population, indicating a need for targeted clinical interventions. Using a multiple logistic stepwise regression model, the study found that factors such as age, marital status, and family income were not significant determinants of awareness levels. This contrasts with existing domestic studies, potentially reflecting differences in regional characteristics, sample sizes, demographic compositions, or statistical methodologies.^[18–20]^ The representativeness of the sample was a key consideration in this study. A stratified random sampling method was employed to ensure a balanced proportion of rural (48.9%) and urban (51.1%) participants, accurately reflecting the demographic distribution of the study area. This methodological approach enhances the reliability of the findings. However, it is important to acknowledge that region-specific socioeconomic and cultural factors may still influence the results. Future studies encompassing more diverse geographic regions are recommended to validate these findings further and improve their generalizability.

Factors such as educational level, occupation, place of residence, and limited dissemination of scientific knowledge were identified as significant risk factors influencing awareness of high-risk HPV infection and cervical diseases among women of childbearing age. Educational level, in particular, reflects an individual’s knowledge base, cognitive abilities, analytical skills, and capacity for information processing.^[21–23]^ Women with higher education levels exhibited better health literacy, greater attention to personal health, and stronger adherence to health-related behaviors. Consequently, they were more likely to seek information on high-risk HPV infection, cervical diseases, and vaccination. In contrast, women with lower educational attainment often faced barriers such as poor working conditions, limited capacity to absorb health-related knowledge, extended working hours, and low income.^[24,25]^ These challenges contributed to a lack of health awareness and inadequate attention to personal health, impeding their ability to learn about HPV infection and cervical disease prevention, which ultimately resulted in poor cognitive outcomes. Occupational and cultural disparities further amplified these differences. Administrative institutions and corporations typically prioritize employee health by providing benefits such as regular physical examinations, which contributed to higher awareness levels among women employed in these sectors. Conversely, women in less formal or unstable employment settings, such as gig workers, had fewer opportunities for accessing health information and services. Place of residence also played a significant role, with urban women and those employed in professional settings having access to more formal and diverse information channels. These advantages translated into more positive attitudes and quicker actions towards preventing and addressing high-risk HPV infections and cervical diseases. Research supports the notion that HPV infection and cervical diseases are influenced by a combination of biological,^[26,27]^ environmental, lifestyle, and behavioral factors. Addressing these disparities through targeted health education and interventions is crucial for improving awareness and prevention efforts among women of childbearing age. Promoting scientific knowledge across diverse groups could play a critical role in addressing these disparities. By enhancing access to information and improving awareness of HPV infection and cervical diseases, women of childbearing age may adopt healthier lifestyle choices, engage in safer sexual behavior, and show increased uptake of HPV vaccination. This shift from passive to active prevention is essential for reducing the burden of cervical diseases and improving overall public health outcomes.

To improve awareness of HPV and cervical diseases among women of childbearing age, several public health interventions should be considered. Internationally, proven health education strategies, such as school-based education programs, community outreach initiatives, and media campaigns, have shown significant impact in increasing knowledge and encouraging HPV vaccination uptake. For example, integrating HPV awareness into school curricula and offering free or subsidized vaccination for adolescents has been an effective strategy in countries like Australia and the United States.^[28,29]^ Local governments could also collaborate with healthcare providers to organize targeted educational campaigns in rural areas, where awareness tends to be lower, using mobile health units or local healthcare centers to disseminate information. Furthermore, strengthening the role of primary healthcare providers in counseling patients about HPV and its risks can promote early detection and prevention. Offering regular screening and vaccination services in workplaces, especially for women in occupations with less access to formal health education, could also improve coverage.

Despite the strengths of this study, several limitations should be acknowledged. First, the study was conducted in a single city (Shijiazhuang), which may limit the generalizability of the findings to other regions with differing socioeconomic and cultural contexts. Second, although a stratified random sampling method was used to balance urban and rural participants, the sample size of 544 women may not fully capture the diversity within the population. Third, the cross-sectional design of the study restricts the ability to establish causal relationships between awareness levels and influencing factors. Finally, self-reported data on awareness and behaviors may be subject to reporting bias, which could affect the accuracy of the findings. Future research should consider expanding the geographic scope, increasing the sample size, and incorporating longitudinal designs to address these limitations and validate the results further.

The prevalence of high-risk HPV infection in the cervix of women of childbearing age in this region remains low, with HPV-16 being the predominant subtype associated with cervical epithelial neoplasia and cervical cancer. Implementing educational initiatives on high-risk cervical HPV infection and cervical diseases among key populations with lower educational backgrounds and in rural areas can enhance awareness and contribute to the prevention and management of high-risk HPV infection and cervical diseases.

## Author contributions

**Conceptualization:** Xiaohui Deng, Yonghong Yang, Xiaoyan Wen, Zhengyan Dai.

**Data curation:** Xiaohui Deng, Yonghong Yang, Xiaoqing Pang, Xiaoyan Wen, Zhengyan Dai.

**Formal analysis:** Yonghong Yang, Xiaoqing Pang, Xiaoyan Wen, Zhengyan Dai.

**Investigation:** Xiaohui Deng, Yonghong Yang, Xiaoqing Pang, Xiaoyan Wen, Zhengyan Dai.

**Methodology:** Xiaohui Deng, Yonghong Yang, Xiaoqing Pang, Xiaoyan Wen, Zhengyan Dai.

**Validation:** Xiaoyan Wen, Zhengyan Dai.

**Writing – original draft:** Xiaohui Deng, Yonghong Yang, Xiaoqing Pang, Zhengyan Dai.

**Writing – review & editing:** Xiaohui Deng, Yonghong Yang, Xiaoqing Pang, Zhengyan Dai.

## References

[1] Sivars L Palsdottir K Crona Guterstam Y Falconer H Hellman K Tham E. The current status of cell-free human papillomavirus DNA as a biomarker in cervical cancer and other HPV-associated tumors: a review. Int J Cancer. 2023;152:2232–42. 36274628 10.1002/ijc.34333

[2] Garolla A Graziani A Grande G Ortolani C Ferlin A. HPV-related diseases in male patients: an underestimated conundrum. J Endocrinol Invest. 2024;47:261–74. 37770654 10.1007/s40618-023-02192-3 PMC10859347

[3] Katirachi SK Grønlund MP Jakobsen KK Grønhøj C von Buchwald C. The prevalence of HPV in oral cavity squamous cell carcinoma. Viruses. 2023;15:451. 36851665 10.3390/v15020451 PMC9964223

[4] Einstein MH Baron M Levin MJ. Comparative immunogenicity and safety of human papillomavirus (HPV)-16/18 vaccine and HPV-6/11/16/18 vaccine: follow-up from months 12–24 in a Phase III randomized study of healthy women aged 18–45 years. Human Vaccines. 2011;7:1343–58. 22048173 10.4161/hv.7.12.18281 PMC3338932

[5] Mudhigeti N Kalawat U Hulikal N Racherla RG. E6–E7 based nested multiplex PCR assay for genital HPV detection and simultaneous typing of 15 high and low-risk HPV types. Indian J Med Microbiol. 2022;40:18–23. 34871707 10.1016/j.ijmmb.2021.11.009

[6] Cambrea SC Aschie M Resul G. HPV and HIV coinfection in women from a southeast region of Romania-PICOPIV study. Medicina (Kaunas, Lithuania). 2022;58:760. 35744023 10.3390/medicina58060760 PMC9231193

[7] Yang X Li Y Tang Y. Cervical HPV infection in Guangzhou, China: an epidemiological study of 198,111 women from 2015 to 2021. Emerg Microbes Infect. 2023;12:e2176009. 36744409 10.1080/22221751.2023.2176009 PMC9936994

[8] Dai Z Si M Su X. Willingness to human papillomavirus (HPV) vaccination and influencing factors among male and female university students in China. J Med Virol. 2022;94:2776–86. 34825712 10.1002/jmv.27478 PMC9299831

[9] Yin G Zhang Y Chen C Ren H Guo B Zhang M. Have you ever heard of human papillomavirus (HPV) vaccine? The awareness of HPV vaccine for college students in China based on meta-analysis. Human Vaccin Immunother. 2021;17:2736–47. 10.1080/21645515.2021.1899731 PMC8475627 33787459

[10] Wadhwa V Chadha K Doshi HN Lad A Bajpai R. Comparison of HPV DNA detection platforms. Indian J Med Microbiol. 2023;41:39. 36870747 10.1016/j.ijmmb.2022.12.007

[11] Wang HY Lee D Park S. Diagnostic performance of HPV E6/E7 mRNA and HPV DNA assays for the detection and screening of oncogenic human papillomavirus infection among woman with cervical lesions in China. Asian Pacific J Cancer Prev. 2015;16:7633–40. 10.7314/apjcp.2015.16.17.7633 26625774

[12] Zhan X Zhou J Jiang Y. DNA tetrahedron-based CRISPR bioassay for treble-self-amplified and multiplex HPV-DNA detection with elemental tagging. Biosens Bioelectron. 2023;229:115229. 36947920 10.1016/j.bios.2023.115229

[13] Zagorianakou N Mitrogiannis I Konis K Makrydimas S Mitrogiannis L Makrydimas G. The HPV-DNA test in pregnancy: a review of the literature. Cureus. 2023;15:e38619. 37284358 10.7759/cureus.38619 PMC10240385

[14] Luo LP He P Liu QT. Prevalence and genotype distribution of HPV infection among 214,715 women from Southern China, 2012–2018: baseline measures prior to mass HPV vaccination. BMC Infect Dis. 2021;21:328. 33827456 10.1186/s12879-021-06019-5 PMC8028771

[15] Roman BR Aragones A. Epidemiology and incidence of HPV-related cancers of the head and neck. J Surg Oncol. 2021;124:920–2. 34558067 10.1002/jso.26687 PMC8552291

[16] Lechner M Liu J Masterson L Fenton TR. HPV-associated oropharyngeal cancer: epidemiology, molecular biology and clinical management. Nat Rev Clin Oncol. 2022;19:306–27. 35105976 10.1038/s41571-022-00603-7 PMC8805140

[17] Lin Y Lin WY Lin TW. Trend of HPV molecular epidemiology in the post-vaccine era: a 10-year study. Viruses. 2023;15:2015. 37896791 10.3390/v15102015 PMC10612033

[18] Chaturvedi AK Engels EA Pfeiffer RM. Human papillomavirus and rising oropharyngeal cancer incidence in the United States. J Clin Oncol. 2011;29:4294–301. 21969503 10.1200/JCO.2011.36.4596 PMC3221528

[19] Stanley M. Realities of alternative HPV vaccination schedules. Salud Publica Mex. 2018;60:617–20. 30699265 10.21149/10190

[20] Sinisgalli E Bellini I Indiani L. HPV vaccination for boys? A systematic review of economic studies. Epidemiol Prev. 2015;39(4 Suppl 1):51–8. 26499416

[21] Reis DRA Medeiros-Fonseca B Costa JM. HPV infection as a risk factor for atherosclerosis: a connecting hypothesis. Med Hypotheses. 2020;144:109979. 32570162 10.1016/j.mehy.2020.109979

[22] Apaydin Arikan E Aydemir L Ulusan M Yilmazbayhan D Ozluk Y. High-risk HPV does not appear to be an important risk factor for sinonasal carcinomas in Turkish population: a tertiary center experience. Int J Surg Pathol. 2023;31:124–36. 35404169 10.1177/10668969221091590

[23] Han J Waller JL Colombo RE. Incidence and risk factors for HPV-associated cancers in women with end-stage renal disease. J Investig Med. 2020;68:1002–10. 10.1136/jim-2019-001262 32503931

[24] Nielsen A Kjaer SK Munk C Iftner T. Type-specific HPV infection and multiple HPV types: prevalence and risk factor profile in nearly 12,000 younger and older Danish women. Sex Transm Dis. 2008;35:276–82. 18091564 10.1097/OLQ.0b013e31815ac5c7

[25] Abdullahi LH Kagina BM Ndze VN Hussey GD Wiysonge CS. Improving vaccination uptake among adolescents. Cochrane Database Syst Rev. 2020;1:CD011895. 31978259 10.1002/14651858.CD011895.pub2 PMC6984618

[26] Durmisević S Serdarević-Durmisević J. Life style based on a system of classical moral values in preventing drug addiction as a risk factor for HIV infection. Med Arh. 2004;58:306–8. 15628258

[27] Semple SJ Strathdee SA Zians J Patterson TL. Life events and sexual risk among HIV-negative, heterosexual, methamphetamine users. J Sex Res. 2010;47:355–63. 19513922 10.1080/00224490903015843 PMC2888692

[28] Joshi S Anantharaman D Muwonge R. Evaluation of immune response to single dose of quadrivalent HPV vaccine at 10-year post-vaccination. Vaccine. 2023;41:236–45. 36446654 10.1016/j.vaccine.2022.11.044 PMC9792650

[29] Tan S Wang S Zou X. Parental willingness of HPV vaccination in Mainland China: a meta-analysis. Hum Vaccin Immunother. 2024;20:2314381. 38385893 10.1080/21645515.2024.2314381 PMC10885179

